# Chloroquine Treatment Enhances Regulatory T Cells and Reduces the Severity of Experimental Autoimmune Encephalomyelitis

**DOI:** 10.1371/journal.pone.0065913

**Published:** 2013-06-14

**Authors:** Rodolfo Thomé, Adriel S. Moraes, André Luis Bombeiro, Alessandro dos Santos Farias, Carolina Francelin, Thiago Alves da Costa, Rosária Di Gangi, Leonilda Maria Barbosa dos Santos, Alexandre Leite Rodrigues de Oliveira, Liana Verinaud

**Affiliations:** 1 Department of Structural and Functional Biology, University of Campinas, Campinas, São Paulo, Brazil; 2 Department of Genetics, Evolution and Bioagents, University of Campinas, Campinas, São Paulo, Brazil; Charité-University Medicine Berlin, Germany

## Abstract

**Background:**

The modulation of inflammatory processes is a necessary step, mostly orchestrated by regulatory T (Treg) cells and suppressive Dendritic Cells (DCs), to prevent the development of deleterious responses and autoimmune diseases. Therapies that focused on adoptive transfer of Treg cells or their expansion *in vivo* achieved great success in controlling inflammation in several experimental models. Chloroquine (CQ), an anti-malarial drug, was shown to reduce inflammation, although the mechanisms are still obscure. In this context, we aimed to access whether chloroquine treatment alters the frequency of Treg cells and DCs in normal mice. In addition, the effects of the prophylactic and therapeutic treatment with CQ on Experimental Autoimmune Encephalomyelitis (EAE), an experimental model for human Multiple Sclerosis, was investigated as well.

**Methodology/Principal Findings:**

EAE was induced in C57BL/6 mice by immunization with myelin oligodendrocyte glycoprotein (MOG_35–55_) peptide. C57BL/6 mice were intraperitoneally treated with chloroquine. Results show that the CQ treatment provoked an increase in Treg cells frequency as well as a decrease in DCs. We next evaluated whether prophylactic CQ administration is capable of reducing the clinical and histopathological signs of EAE. Our results demonstrated that CQ-treated mice developed mild EAE compared to controls that was associated with lower infiltration of inflammatory cells in the central nervous system CNS) and increased frequency of Treg cells. Also, proliferation of MOG_35–55_-reactive T cells was significantly inhibited by chloroquine treatment. Similar results were observed when chloroquine was administrated after disease onset.

**Conclusion:**

We show for the first time that CQ treatment promotes the expansion of Treg cells, corroborating previous reports indicating that chloroquine has immunomodulatory properties. Our results also show that CQ treatment suppress the inflammation in the CNS of EAE-inflicted mice, both in prophylactic and therapeutic approaches. We hypothesized that the increased number of regulatory T cells induced by the CQ treatment is involved in the reduction of the clinical signs of EAE.

## Introduction

The modulation of the immune system is a necessary process to prevent the development of deleterious immune response and autoimmune diseases. Several mechanisms were developed to restrain exacerbated activation of the immune system against self-antigens which includes the central and peripheral tolerance [1]–[3]. Thymocytes, the lymphocytes inside the thymus, are “tamed” to recognize auto-antigens and respond to non-self-antigens within the thymic environment, in a network of soluble molecules, cell-cell and cell-extracellular matrix interactions [4]–[6]. In periphery, natural arising regulatory T (Treg) cells act inhibiting the activation of self-reactive lymphocytes through cell contact, secretion of anti-inflammatory cytokines and modulation of professional antigen presenting cells, like dendritic cells (DCs) [3], [7], [8]. It was previously shown that a reduction in number and function of Treg cells is associated with autoimmune diseases [9]–[11], and failure to express the nuclear transcriptional factor Foxp3 results in human X-linked IPEX (Immunodysregulation Polyendocrinopathy and Enteropathy) and mouse *scurfy*, both severe poly-autoimmune disease syndromes [12], [13].

Adoptive transfer of Treg cells has proven to be a useful tool to reduce inflammatory diseases, such as human graft versus host disease [14], experimental diabetes [15], experimental autoimmune hepatitis [16], experimental arthritis [17] and experimental autoimmune encephalomyelitis [18]. Therefore, therapies that promote the expansion of regulatory T cells are desirable in order to reduce the overall chronic inflammation observed in most autoimmune diseases. Chloroquine (CQ), an anti-malarial drug, has proven to exert some anti-inflammatory effects through the down-regulation of Tumor Necrose Factor- alpha (TNF-α) production and signaling in macrophages [19], [20], as well as the cytokine pattern production [21]. Yet, the administration of chloroquine prevented the onset of graft-versus-host disease in a mouse model [22]. Treatment with chloroquine together with other immunosuppressive drugs resulted in amelioration of the clinical manifestations in rheumatoid arthritis patients [23]. It is not clear the precise mechanism triggered by chloroquine, but several evidences suggest that chloroquine acts as a weak base by both pH-dependent and –independent mechanisms [24]–[26].

Experimental Autoimmune Encephalomyelitis (EAE) is the most studied experimental model for Multiple Sclerosis, which is originated after immunization of susceptible mice with myelin-associated proteins in an inflammatory context. Activated T cells migrate into the Central Nervous System (CNS) and initiate a robust inflammatory response [27]–[29]. Thus far, the treatment for MS is based on high cost medicine and more recently on the administration of monoclonal antibodies [30]–[32]. So, the search for adjunctive therapies is of great value in the field of autoimmunity treatment, especially those that increase the frequency or function of regulatory T cells. In this sense, chloroquine is a cheap and well-tolerated drug, with some described effects on inflammatory conditions. However, the mechanisms used by chloroquine and whether regulatory T cells are involved in the immunomodulation as well as whether this drug can reduce the clinical signs of EAE, remain obscure.

In this context, we aimed to investigate if the administration of chloroquine alters the frequency of regulatory T cells and dendritic cells in the periphery of the immune system and if the treatment with CQ could ameliorate the clinical signs of EAE. We found that CQ treatment provoked an increase in the frequency of Treg cells and reduced DCs numbers in the spleen. When CQ was administrated both prophylactic and therapeutically mice developed mild clinical score of EAE and this was accompanied by a reduced infiltration of inflammatory cells to the CNS. An increase in Treg cells number and in secretion of immunomodulatory cytokines was observed as well. The data obtained here strongly suggest that chloroquine may become a useful adjunct in the treatment of multiple sclerosis.

## Materials and Methods

### Mice

Six-to-eight week-old female C57BL/6 mice from the Multidisciplinary Center for Biological Research, University of Campinas, were used in this study. Mice were kept in *specific-pathogen free* conditions, in a controlled temperature and photoperiod environment, with free access to autoclaved food and water throughout the experiment. All protocols involving laboratory animals were approved and performed in accordance with the guidelines of the State University of Campinaś Committee on the Use and Care of Animals (Comissão de Ética no Uso de Animais – CEUA, # 2687-1).

### Chloroquine Treatment

Groups of mice (n = 7) were created aiming the test for ideal, non-toxic chloroquine (Chloroquine diphosphate salt, Sigma-Aldrich, Brazil) concentration. The concentrations tested were 3, 5 and 10 mg.kg^−1^. The 100 mg/kg dose was found to be lethal. Animals of each group received chloroquine via i.p. (200 µL/mice) for five consecutive days. Control mice were injected with diluent solution (Phosphate-Buffered Saline 0,02 M pH 7,2). Three days after the last dose, mice were killed and splenic cells were collected and assayed for cellular population analysis in the presence of concanavalin-A (2,5 µg/mL). Mice survival and spleen cellularity were evaluated as well.

### EAE Induction, Evaluation and Chloroquine Treatment

EAE was induced and evaluated in mice according to a previous published paper [33]. Briefly, each mouse was injected with 100 µg MOG_35–55_ (MEVGWYRSPFSRVVHLYRNGK, RheaBiotec, Brazil) emulsified with Complete Freunds Adjuvant (CFA, Sigma-Aldrich, USA). 200 ηg *Pertussis* toxin (Ptx, Sigma-Aldrich, USA) was administrated via i.p. at 0 and 48 h after MOG_35–55_ inoculation. Weight changes and clinical signs were followed and graded daily according to a score method, where 0: no sign, 1: flaccid tail, 2: hind limbs weakness, 3: hind limbs paralysis, 4: hind paralysis and fore limbs weakness, 5: full paralysis/dead. An intermediate non-toxic concentration (5 mg/kg/day) of chloroquine was used for EAE treatment (five consecutive days, via i.p.). For prophylactic approach, EAE was induced three days after the last dose of CQ (5 mg.kg^−1^), and for therapeutic approach, mice received the CQ treatment after the onset of EAE (day 10^th^ after immunization with neuro-antigens). Fourteen (prophylactic approach) and thirty (therapeutically approach) days after antigen challenge mice were killed spinal cords were removed and snap frozen; 12 um thin slices were made in cryostat and stained with haematoxylin and eosin (H&E).

### Isolation of Treg Cells (CD4^+^CD25^+^) and Transfer Experiments

Naïve C57BL/6 mice were treated with chloroquine as described above and three days after the last dose spleen cells were collected and CD4^+^CD25^+^ cells were isolated by magnetic beads following manufactureŕs recommendations (CD4^+^CD25^+^ Regulatory T Cell Isolation Kit; Miltenyi Biotec., USA). 5×10^5^ Treg cells per mouse were adoptively transferred (via i.v.) to EAE mice at the onset of disease (10 days after immunization). As control, EAE mice received equal numbers of CD4^+^CD25^−^ cells at the same time point. EAE induction and evaluation was performed as described above.

### Lymphoproliferative Response and Cytokine Dosage

Splenic cells were aseptically collected from mice after 10 and 30 days of antigen challenge for prophylactic and therapeutic approaches, respectively, and after 16 days for Treg cells transfer experiments. Single cell suspensions were stained with Carboxyfluorescein succinimidyl ester (CFSE, Sigma-Aldrich, USA) following the manufactureŕs instructions. Cells (5×10^5^/well) were diluted in RPMI 1640 media supplemented with Fetal Calf Serum (FCS;10% vol/vol), guaramicine (50 ug/mL), 2-Mercaptoethanol (2 mM) and myelin oligodendrocyte glycoprotein peptide (MOG_35–55;_20 ug/mL), plated in flat-bottom plates and incubated at 5% CO_2_ and 37°C for 96 h. After the incubation period, cells were stained with PercPCy5-conjugated anti-CD3 antibodies and fixed in 1% paraformaldehyde prior to flow cytometer analysis. CFSE^low^CD3^+^ cells were considered proliferating T cells. Culture supernatants were collected and assayed for cytokines (IL-4, IL-6, IL-10, IL-17, IFN-γ and TNF-α) secretion using the Cytometric Bead Array (CBA, BD Biosciences, USA) according to manufactureŕs instructions.

### Analysis of Cellular Infiltration in the CNS

Fourteen days after EAE induction (in the prophylactic approach) and thirty days after EAE induction (in the therapeutic approach), mice were anesthetized, perfused with ice cold PBS and half of the spinal cords and brains were removed and stored at −80°C until use for RT-PCR assays; the remaining tissue was prepared for the enrichment of infiltrating leukocytes according to a previously described methodology and analyzed by flow cytometry [34].

### Flow Cytometry

Fluorochrome-conjugated monoclonal antibodies were used to stain leukocytes. Cells were surface stained with anti-CD4/PE-Cy7, anti-CD8/APC, anti-CD3/PercPCy5, CD11c/APC, CD11b/PE, F4-80/APC, TLR-2/PE, TLR-4/PE. For intracellular staining, cells were fixed/permeabilized (fixation/permeabilization buffers) according to manufactureŕs recommendations, later monoclonal anti-Foxp3/APC, IL-10/PE, IFN-γ/PE and IL-17/APC were added to cells. Isotype controls were used as well. All antibodies were purchased from eBioscience (USA). Preparations were acquired with a Gallios flow cytometer (Becman Coulter, USA) and data analyzed using FlowJo 7.6 (Tree Star Inc., USA).

### RT-PCR Assays

Frozen tissues were used for RNA extraction using Trizol (Invitrogen, USA) and cDNA synthesis according to the manufactureŕs recommendations (Applied Biosystems, USA). Expression of IL-10 (Mm00439614_m1), IL-17 (Mm00439618_m1), IFNg (Mm01168134_m1), FOXP3 (Mm00475162_m1) and RAR-related orphan receptor C (RORc) (Mm01261022_m1) were analyzed in comparison to GAPDH (Mm99999915_g1, housekeeping gene) levels. RT-PCR reactions were performed using Taqman reagents according to manufactureŕs recommendations (Applied Biosystems, USA).

### Statistical Analysis

Clinical score comparisons between control and experimental groups were done by Two-Way ANOVA and post-tested with Bonferroni. Other analyses among two and three (or more) groups were carried out with Students t test and One-Way ANOVA, respectively. Results are expressed as mean ± standard error mean (SEM) and p<0,05 value were defined as significant.

## Results

### Increased Frequency of Regulatory T cells and Reduced Percentage of Dendritic Cells after Chloroquine Treatment

**Figure 1:**
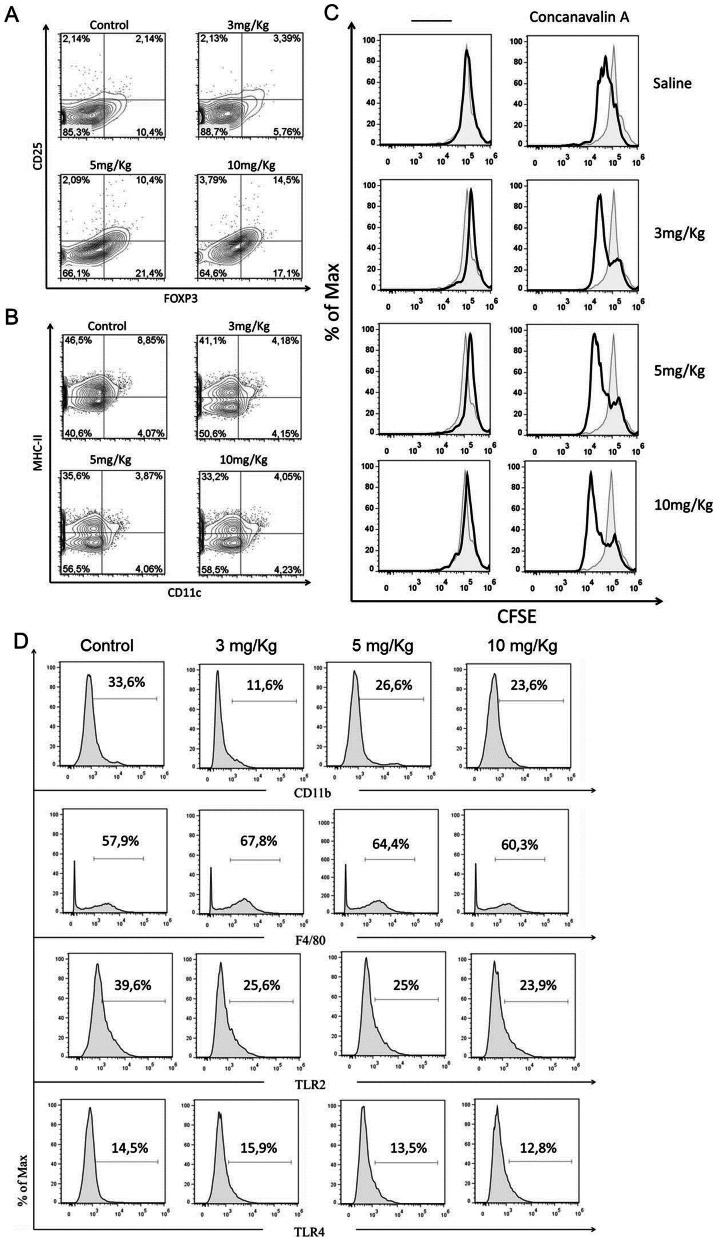
Chloroquine administration alters the frequency of regulatory T (Treg) cells and dendritic cells (DCs), but not the proliferative capability of T cells. Briefly, mice were treated with chloroquine via i.p. for five consecutive days. Three days after the last dose mice were killed and splenic cells were analyzed by flow cytometry. Increased numbers of Treg cells (A) and reduced frequency of DCs (B) was found in mice treated with chloroquine when compared to the control group. In addition, splenic T cells proliferative response was not altered in the presence of concanavalin-A (C). Subpopulations of leukocytes showed slight changes when compared to control subjects (D). Results are representative of three independent experiments.

Naïve mice were treated with CQ at different dosages for five consecutive days and the cellular subsets were evaluated three days after the last dose of drug administration. Along with reduction in the total splenic cells number at higher doses (data not shown), our data showed that CQ treatment increased the numbers of regulatory T cells whereas the frequency of dendritic cells was reduced (Figure 1A and 1B, respectively). In order to evaluate whether CQ treatment promoted functional alterations in T cells, splenic lymphocytes from CQ treated-mice were cultured in the presence of concanavalin-A (Con-A) for 72 h. As depicted in figure 1C, the CQ treatment did not alter the proliferation capacity of T cells. Other subpopulations of leukocytes were also analyzed but only a slight change in the frequency of these cells was noticed compared to the control group (Figure 1D).

### Chloroquine Treatment Reduces the Clinical Evolution and Infiltration of the CNS in EAE Mice

**Figure 2:**
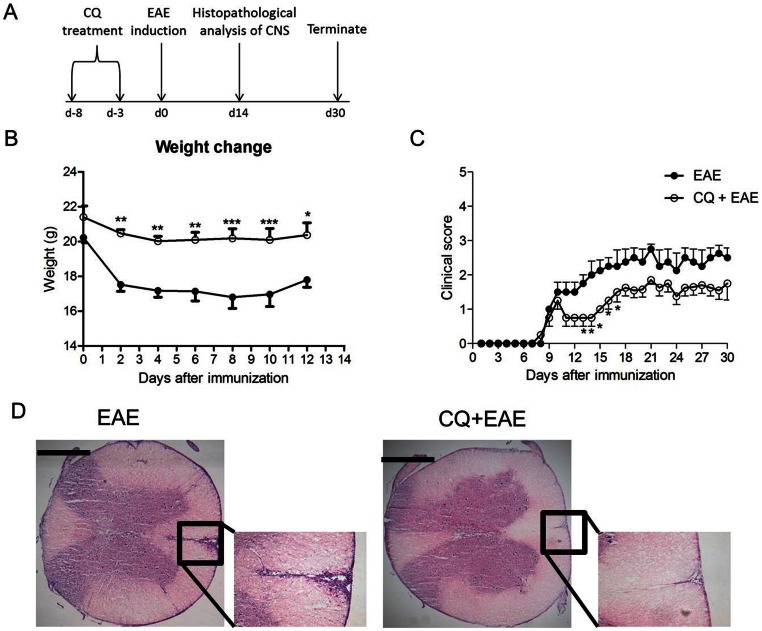
CQ treatment reduces the severity of EAE. (A) Briefly, mice were treated with chloroquine for five consecutive days and three days after the last dose of the drug EAE was induced through the administration of MOG_35–55_ peptide emulsified in CFA and two injections of Ptx (200 ng/animal) at 0 and 48 h post antigen inoculum. (B and C) The weight change and clinical score were checked routinely. Results are expressed as mean ± SEM for at least five animals. p<0,01 (**) and p<0,005 (***). (D) At 14 days, mice were killed and spinal cords were prepared for histological analysis (H&E stained) to evaluate cellular infiltration in the CNS. The figures are representative of at least three independent experiments performed in different days. Bar: 500 µm.

An increase in regulatory T cells pool is associated with mild inflammation, whereas reduced dendritic cell numbers may impair proper antigen presentation to T cells, thus dampening adaptive immune response. In this context, the next goal was to determine whether prophylactic CQ administration was capable of modulating the course and severity of EAE. Hence, mice were subjected to CQ treatment (5 mg/kg/day) for five consecutive days, and three days after the last dose EAE was induced (Figure 2A) and the development of the disease accompanied daily.

Mice that received CQ prior to EAE induction showed a significant reduction in weight loss compared with PBS-treated animals. Accordingly, the treatment was also capable to delay disease severity course (Figures 2B and 2C). As leukocytes infiltration in the CNS is directly associated with the severity of disease, we aimed to investigate whether the CQ treatment had altered brain inflammation. PBS- and CQ-treated EAE mice were killed and spinal cords were removed and stained with H/E. Corroborating results mentioned above, CQ treated-mice presented lower leukocytes infiltration in the CNS (Figure 2D). Overall, CQ administration was able to ameliorate the clinical course of EAE, most probably, because of the reduced cellular infiltration in the CNS.

**Figure 3:**
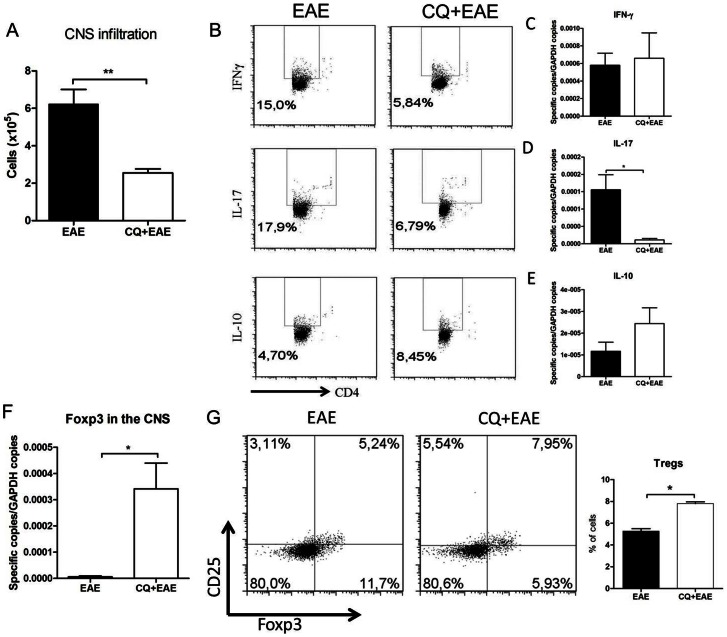
Analysis of the cellular infiltration of the CNS show reduced IFN-γ and IL-17 producing cells in CQ treated EAE mice. (A) CQ treated-mice presented reduced infiltration of inflammatory cells. (B) The percentage of IFN-γ- and IL-17-producing cells infiltrating the brain was reduced while the frequency of IL-10- producing cells was found augmented in brain of mice treated with CQ. (C, D and E) Gene expression of IFN-γ, IL-17 and IL-10 in the CNS followed the same pattern, respectively. (F) The expression of FOXP3 was evaluated in the CNS by RT-PCR. (G) The frequency of CD25^+^Foxp3^+^ cells was evaluated in spleens of mice. Results are representative of two independent experiments and are expressed as mean ± SEM for at least five animals. p<0,05 (*) and p<0.01 (**).

We next examined the profile of leukocytes that infiltrated the CNS of CQ treated-EAE mice. For that purpose brains and spinal cords were collected, minced and cellular suspensions were prepared and analyzed as described in M&M section. Our results show that lymphocytes managed to overcome the blood-brain barrier and infiltrated the CNS of EAE mice, both of PBS- and CQ-treated groups. However, the number of infiltrating lymphocytes was significant reduced in CQ treated-mice compared to the control subjects (Figure 3A). Interestingly, the pattern of infiltrating cells in CQ-treated group was quite different from control EAE group. CQ treated-mice showed significant reduction in interleukin (IL)-17A- and interferon-gamma (IFN-γ)-producing cells and a significant increase of IL-10-producing cells in the CNS (Figure 3B). Also, the relative gene expression analyses have showed decreased pattern for IL-17 and IFN-γ and up-regulated pattern for IL-10 (Figure 3C, 3D and 3E, respectively).

As Treg cells number were increased in normal mice upon CQ treatment and an augmented frequency of this population is correlated with suppression of EAE, we aimed to assess the incidence of Treg cells in spleen and the Foxp3 gene expression in the spinal cords of EAE mice fourteen days after induction of the disease. Corroborating our results, the expression of Foxp3 was found significantly augmented in CQ treated-mice (Figure 3F). In the periphery of the immune system, it was observed that EAE mice that received CQ had increased Treg cell numbers compared with the PBS treated-group (Figure 3G). These data indicate that the reduction in EAE severity observed in CQ-treated mice correlates with the increase in Treg cells number both in the CNS and the periphery.

### Administration of Chloroquine Suppresses the Ag-specific Proliferation and Changes the Cytokine Production Pattern

**Figure 4:**
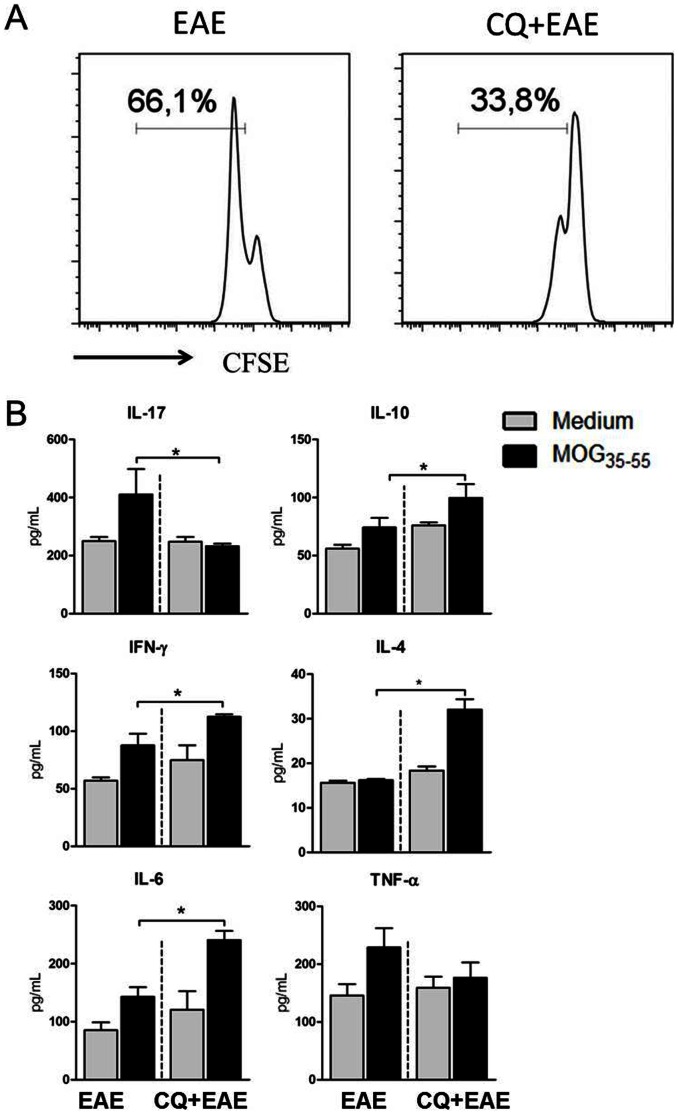
Chloroquine treatment reduces the Ag-specific proliferation of T cells in the spleen and the cytokine production profile. After 14 days of immunization with MOG_35–55_ peptide, mice were killed and CFSE-stained splenocytes were cultivated in the presence of MOG_35–55_ for 96 h. (A) The proliferation was calculated in the CFSE^low^CD3^+^ cells. Figures presented are representative of three independent experiments. (B) At the end of the culture period the supernatants were collected and assayed for the detection of cytokines using cytometric beads assay. Results are expressed as mean ± SEM for at least five animals. p<0,05 (*).

**Figure 5:**
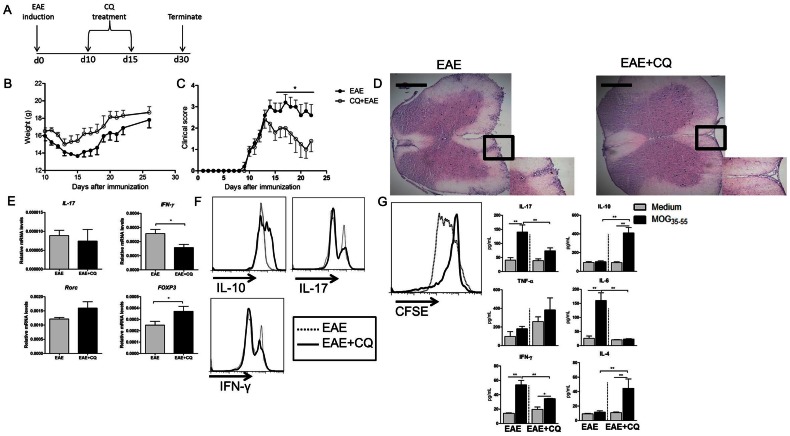
Chloroquine administration after the onset of EAE reduces the clinical signs of the disease. (A) CQ was administrated ten days after immunization with MOG_35–55_. (B) and (C) Animals were accompanied for weight changes and clinical score. (D) The spinal cords were removed at day 30 and 10 µm slices were stained with HE for detection of infiltrating cells in the CNS. Figures are representative of at least six mice. Bar: 500 µm. (E) Gene expression of IL-17, IFNγ, Foxp3 and RORc in the CNS was evaluated. (F) The infiltrating cells in the CNS were collected and stained for flow cytometric characterization of IL-10, IL-17 and IFN-γ production. (G) Spleen cells from EAE mice, CQ- and PBS-treated mice, were removed and CFSE-stained cells were cultivated (5×10^5^/well) in the presence of MOG_35–55_ peptide (20 µg/mL) for 96 h. The dye decay and cytokine production were measured by flow cytometry. Results are representative of three independent experiments and are expressed as mean ± SEM for at least five animals. * p<0.05.

Considering that an increase in Treg and IL-10-producing cells may correlate with the reduced clinical signs of EAE, and that the antigen-specific cellular immune response is the cause of the disease in mice, we next evaluated whether peripheral encephalitogenic lymphocytes from CQ treated-mice proliferate in the presence of MOG_35–55_. For that purpose, splenic leukocytes derived from mice after ten days of immunization with neuro-antigen were collected and put in culture in the presence of MOG_35–55_ for 96 h. Our data show that lymphocytes from CQ-treated mice proliferated significantly less than cells from PBS–treated group (Figure 4A). In the culture supernatants there was also a significant reduction in IL-17 levels, whereas the concentration of IL-10, IL-6, IFN-γ, and IL-4 were found significantly up regulated from CQ-treated mice cells compared to PBS-treated ones. No difference could be observed in the levels of tumor necrosis factor-alpha (TNF-α) between cultures of both groups (Figure 5B).

### Chloroquine Treatment may also be Used after the Onset of EAE with Similar Results

Although CQ prophylactic approach was able to reduce the clinical evolution of EAE, the results might differ when the drug is administrated after disease onset, which corresponds to a more realistic picture for disease treatment. In order to solve this issue, mice were immunized with MOG_35–55_ and 10 days later, after the onset of EAE, CQ treatment was initiated (Figure 5A). Results showed that CQ-treated EAE mice presented a reduction in the weight loss and amelioration of the clinical course of the disease (Figure 5B and 5C, respectively).

EAE develops after the migration of inflammatory cells to the CNS, where they produce pro-inflammatory cytokines and secrete a myriad of enzymes and soluble factors damaging the nervous system. As the treatment started after disease onset, we next evaluated whether the cellular infiltration in the spinal cords of mice was altered. Thirty days after EAE induction spinal cords were collected and analyzed for the presence of leukocytes. We found that CQ treatment provoked a slight reduction in the infiltration of cells to the spinal cords compared with the PBS-treated group (Figure 5D). Although CQ treatment was not able to reduce leukocytes infiltration in the CNS, a significant up-regulation of Foxp3 cells in the spinal cords was observed. The expression of IFN-γ was found significantly down-regulated in the treated group as well.

The expression of IL-17 and Th17 related transcriptional factor RAR-related orphan receptor C (RORc) was not statistically different between the two groups (Figure 5E). Accordingly, the profile of inflammatory cells in the CNS was altered as the frequency of IL-10-producing cells was augmented while the frequency of IFN-γ- and IL-17-producing cells was reduced in the CQ-treated group (Figure 5F). There was also reduction in MOG_35–55_ -specific proliferation of splenocytes from CQ-treated mice compared to control group and IL-17, IL-6, IFN-γ secretion. In contrast, IL-10 and IL-4 production was augmented when cells were cultured in the presence of MOG_35–55_ peptide (Figure 5G).

### Transfer of Chloroquine-elicited Regulatory T cells Reduces EAE

**Figure 6:**
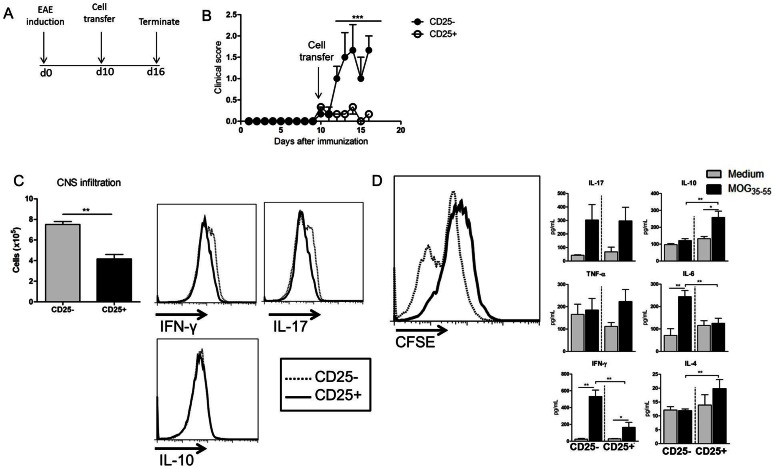
Transfer of CQ-elicited Treg cells reduces the severity of ongoing EAE. (A) Naïve C57BL/6 mice were treated with chloroquine (5 mg/kg/day) for five consecutive days. Three days after the last dose of the treatment, splenic CD4^+^CD25^+^ cells were isolated using magnetic beads and cells (5×10^5^ cells per mouse) were transferred into mice with ongoing EAE (10 days after immunization). As controls, mice received the same number of CD4^+^CD25^−^ cells. (B) The clinical course of the disease was evaluated routinely. (C) The brains and spinal cords were collected and the enriched infiltrating cells were counted. The frequency of IL-17-, IL-10- and IFN-γ-producing cells was analyzed by flow cytometry as well. (D) The spleens were collected and CFSE-stained cells were cultivated in the presence of MOG_35–55_ peptide for 96 h. Dye decay and cytokine production were analyzed by flow cytometry. Results are expressed as mean ± SEM for at least five animals. p<0,05 (*), p<0,01 (**) and p<001 (***).

As we have observed that CQ in homeostatic conditions is able to promote an increase in Treg cells, we decided to investigate whether Treg cells elicited by CQ treatment played a role in the modulation of EAE severity. Then naïve C57BL/6 mice were treated with CQ for five consecutive days (5 mg/kg/day) and their isolated CD4^+^CD25^+^ (Treg) cells were transferred into EAE mice at the disease onset (day 10 after MOG_35–55_ inoculation) (Figure 6A). Results showed that transfer of Treg cells reduced the clinical course of EAE (Figure 6B) compared to CD4^+^CD25^–^recipient EAE mice (Figure 6B). There was also reduction in the leukocytes infiltration in the CNS (Figure 6C). We next characterized the cytokine profile of the infiltrating cells. Mice that received Treg cells at EAE onset had lower frequency of IL-17- and IFN-γ-producing cells in the CNS compared to the control group. The frequency of IL-10 producing cells remained unchanged (Figure 6C).

The MOG_35–55_-specific cellular response in the periphery was evaluated as well. It was observed that splenic cells from EAE mice that received CD25^+^-transferred cells proliferated significantly less than cells from CD25^−^ -transferred-EAE mice (Figure 6D). We aimed to assess whether the pattern of cytokine production in the presence of MOG_35–55_ peptide was altered. Our data showed that there was no statistical difference in the production of IL-17 and TNF-α between the two groups. However, levels of IFN-γ and IL-6 were reduced while an increase in IL-10 and IL-4 secretion was observed in cell cultures from CD25^+^ transferred- mice when compared to control group (Figure 6D).

## Discussion

Autoimmune diseases develop in deregulated immune systems that fail to control chronic inflammation. Although the events that trigger disease development are unknown, multiple sclerosis is an immune mediated syndrome with characteristics of acute and chronic inflammation [35], [36]. Therapies that focus on reestablishing homeostasis and immunomodulation are of great value. Regulatory T cells play an important role in the control of inflammation and suppression of auto-reactive cells [3], [8], [37]. In this context, we found that chloroquine administration provokes an increase in Treg cells frequency in the spleen of normal mice. When administrated, both prophylactic and therapeutically, CQ modulated the course of EAE, an animal model for multiple sclerosis. The transfer of CQ-elicited Treg cells into mice with ongoing EAE promoted a reduction in disease severity as well.

Chloroquine, an anti-malarial agent, was shown to have anti-inflammatory properties. The administration of the drug resulted in impaired iron metabolism and TNF-α production by macrophages [20], [38], as well as altered cytokine secretion profile [19], [39], [40]. It was also shown that chloroquine affects T cell priming to minor MHC complexes and may be used to modulate *graft-versus-host* disease (GVHD) [41]. The mechanisms underlying these effects are not fully understood, but may involve the changes in pH of several intracellular organelles. CQ is a weak base that has tropism for acidic organelles, such as lisossomes [42]. Although it was already shown that CQ raises NKT cell pool [22], to our knowledge, this is the first study to show that chloroquine treatment leads to an increase in regulatory T cell numbers in the periphery as well as a decrease in DC’s.

Therapies that lead to induction of regulatory T cells have provided interesting results in the amelioration of EAE. The ingestion of the lactic acid producing bacteria *Pediococcus acidilactici* led to expansion of Treg cells in the mesenteric lymph nodes of mice resulting in decreased specific cellular response and consequently in EAE score [43]. Oral administration of MOG_35–55_ also resulted in reduced EAE severity through the stimulation of antigen-specific Treg cells [44]. Therefore, we aimed to access whether prior expansion of Treg cells, due to chloroquine administration, could suppress the development of EAE. Mice treated with CQ developed a mild form of the disease, and Treg cells population was found augmented both in spleen and in the CNS. Although these Treg cells emerged before MOG_35–55_ -immunization, the MOG_35–55_ -specific cellular proliferation was reduced, suggesting that the Treg-mediated immune-suppression is antigen-unspecific. Similarly, Ovalbumin-specific regulatory T cells were able to reduce the anti-Type II Collagen responses, promoting reduced clinical signs of collagen-induced arthritis in a by-stander fashion [45], [46]. In cultures of spleen cells in the presence of MOG_35–55_ peptide we observed a change in the pattern of cytokine secretion. The increased IFN-γ, IL-4 and IL-6 production indicates that CQ treatment altered the T cell subsets responsive to the neuro-antigen. These cytokines may be involved in the deviation of the immune response towards neuro-antigens *in vivo* after CQ administration.

Th1 and Th17 cells are important for EAE development. Both cells act synergistically to induce the lesions in the CNS [47], [48], although IFN-γ-producing cells seems to suppress exacerbated disease [49], [50]. Neutralization of IL-17 by antibodies leads to mild disease severity [51]. Thus, suppressing inflammatory cytokines may result in down-modulation of EAE. The treatment with chloroquine also changed the pattern of cytokine secretion of the infiltrating cells in the CNS; the reduction in the IFN-γ and IL-17-producing cells was correlated with mild disease. It was previously published that administration of MOG antigen, by the oral route, resulted in a change of the inflammatory cells in the CNS, and this promoted low disease severity [34]. The same pattern of suppression was recently observed when DNA vaccine was administrated together with Tacrolimus [52]. Also, MOG-DNA vaccination promoted expansion of regulatory T cells in the periphery and Foxp3 expression in the spinal cords of EAE mice, as well as augmented the expression of neuroprotective genes in the CNS [53].

It is of recent concern that regulatory T cells may turn into effector inflammatory cells. It was found that natural arising and periphery induced Treg cells may become Th1 and Th17 cells *in vivo* and *in vitro* [54]–[57]. The events that lead to this conversion are based on the stimulation of the mTOR cascade, which induces the differentiation of Th1 and Th17 cells in inflammatory and lymphopenic conditions [56]. We did not observe this effect in the treatment of ongoing EAE. In fact, our results show that regulatory T cells raised by the CQ treatment were not converted into effector T cells, even at the 30^th^ day after disease onset, as seen by the augmentation of Foxp3 expression and the reduction in IFN-γ production. So, the treatment with chloroquine of established EAE resulted in reduction of EAE suggesting that a long-lasting immunomodulation can be achieved with this therapy. When CQ-elicited Treg cells are transferred to mice with ongoing EAE, the disease severity was reduced. The cellular response towards neuro-antigens in the periphery was contained and the pattern secretion of cytokines was altered as well. Transfer of CQ-Treg cells also reduced the infiltration of cell into the CNS, although the frequency of IL-10-producing cells was unaltered, which is distinct from the data observed with CQ treatment. The reduced dendritic cells number after CQ therapy may favor the higher suppression profile of CQ treatment over CQ-Treg cells transfer experiments. These data indicate that the amelioration of EAE after CQ treatment is a result of Treg-dependent and -independent mechanisms.

Other anti-malarial drugs are being tested in experimental models of inflammation. Recently, it was published that artimisinin derivative, dihydroartemisinin (DHA), promoted suppression on EAE course [58]. The effects observed were dependent on Treg stimulation in the periphery. The authors showed that the mammalian target of rapamycin (mTOR) signaling cascade was attenuated in T cells [58], which also inhibits Th1 and Th17 differentiation [56]. The hypothesis that CQ treatment interferes with mTOR cascade was not investigated here. However, recent reports demonstrated that chloroquine targets the mTOR pathway in cancer cells inducing cell death [59]. We do not discard the possibility that chloroquine may influence the mTOR pathway promoting regulatory T cells and decreasing the frequency of Th1 and Th17 cells. Other studies must be conducted in order to define the precise mechanism by which chloroquine stimulates Treg cells expansion or differentiation.

It was noticed that chronic abuse of chloroquine (cumulative ingestion of over 1000 g) in rheumatoid patients results in ocular toxic effects that may lead to blindness [60]–[62]. However, proper control of CQ consumption (3–5 mg/kg/day) reduced the incidence of retinopathy to 0,3–2% [63]–[65]. The dose used in this study is acceptable within the dose range for human treatment to produce less collateral effects. But, we highlight that the treatment period in this study was brief. So, other studies must be conducted to assess the efficacy of this treatment in human multiple sclerosis.

The results presented herein indicate that chloroquine reduces the clinical course of EAE. We propose that this effect is due to the expansion of regulatory T cells in the spleen, which reduces the specific cellular response in the periphery. CQ may also affect other T cell subtypes that contribute to the reduced EAE severity. Interestingly, Treg cells also migrate to the CNS to reduce local inflammation and promote protection of the nervous system. Taken together, our data suggest that chloroquine may be a potential drug to be used as an adjunctive therapy in the treatment of multiple sclerosis.
